# Hyperendemic malaria transmission in areas of occupation-related travel in the Peruvian Amazon

**DOI:** 10.1186/1475-2875-12-178

**Published:** 2013-05-31

**Authors:** Benjamin S Parker, Maribel Paredes Olortegui, Pablo Peñataro Yori, Karin Escobedo, David Florin, Silvia Rengifo Pinedo, Roldan Cardenas Greffa, Luis Capcha Vega, Hugo Rodriguez Ferrucci, William K Pan, Cesar Banda Chavez, Joseph M Vinetz, Margaret Kosek

**Affiliations:** 1 Johns Hopkins Bloomberg School of Public Health, 615 N Wolfe Street, Room E5545, Baltimore, Maryland 21205, USA; 2 Asociación Benéfica Prisma, Calle Ramirez Hurtado 622, Iquitos, Peru; 3 US Naval Medical Research Center Unit-6, Iquitos, Peru; 4 US Naval Medical Research Center Unit-6, Lima, Peru; 5 Ministry of Health, Av 28 de Julio s/n, Punchana, Loreto, Peru; 6 Nicholas School of the Environment, Duke University, 310 Tent Drive, Room 227, Durham, NC, USA; 7 Division of Infectious Diseases, Department of Medicine, University of California San Diego, La Jolla, CA, USA

**Keywords:** Malaria, Entomology, Transmission, *Anopheles darlingi*, Sporozoite rate, Entomological inoculation rate, Amazon, Peru

## Abstract

**Background:**

*Plasmodium vivax* and *Plasmodium falciparum* cause a significant illness burden in Peru. Anopheline indices for populated communities in the peri-Iquitos region of Loreto have been reported to be remarkably low, with entomological inoculation rates (EIR) estimated at one to 30 infective bites per year based on a few studies in close proximity to the urban centre of Iquitos and surrounding deforested areas. Local reports suggest that a large number of the reported cases are contracted outside of populated communities in undeveloped riverine areas frequented by loggers and fishermen.

**Methods:**

To better understand vectorial capacity in suspected high malaria transmission zones in a rural district near Iquitos, Peru, mosquito collections were conducted at different points in the seasonality of malaria transmission in 21 sites frequented by occupational labourers. Prevalence of *Plasmodium* spp in vectors was determined by circumsporozoite protein ELISA on individual mosquitoes. Slide surveillance was performed for humans encountered in the zone.

**Results:**

In total, of 8,365 adult female mosquitoes examined, 98.5% were identified as *Anopheles darlingi* and 117 (1.4%) tested positive for sporozoites (*P. falciparum, P. vivax* VK210 or *P. vivax* VK247). Measured human biting rates at these sites ranged from 0.102 to 41.13 bites per person per hour, with EIR values as high as 5.3 infective bites per person per night. Six percent of the 284 blood films were positive for *P. vivax* or *P. falciparum*; however, 88% of the individuals found to be positive were asymptomatic at the time of sampling.

**Conclusions:**

The results of this study provide key missing indices of prominent spatial and temporal heterogeneity of vectorial capacity in the Amazon Basin of Peru. The identification of a target human subpopulation as a principal reservoir and dispersion source of *Plasmodium* species has important implications for vaccine development and the delivery of effective targeted malaria control strategies.

## Background

The history of malaria in Peru shows an expansion of malaria incidence over the past half-century following the eradication campaign of the 1960s when the malaria case burden in Peru fell to 1,500 cases reported nationwide in 1965, and remained at similar levels for approximately 20 years [1,2]. In the Department of Loreto, the presence of *Plasmodium falciparum* first began to be reported between 1988 and 1991, followed shortly afterward by a malaria epidemic in the mid-1990s [3]. Reported cases of malaria nationwide in Peru reached a peak of 85,137 cases in 2005 [4], and in 2008 Peru reported the third highest number of malaria cases in the Americas, exceeded only by Brazil and Colombia [5]. Between 2005 and 2011, the nationwide case burden showed a steady decline, but data collected up to the 52nd epidemiological week of 2012 showed a slight malaria resurgence [6], breaking this trend. Of the cases in Peru, *Plasmodium vivax* remains more prevalent than *P. falciparum,* accounting for 27,523 of the malaria cases reported in 2012 (up to epidemiological week 52), approximately seven times the number of *P. falciparum* cases reported during the same period [6].

Accounting for approximately one-quarter of the geographical size of Peru, the Department of Loreto was reported to have a population of 942,457 inhabitants in 2007 and represents a large portion of the malaria case burden within Peru [7]. Up to the 52nd epidemiological week of 2012, Loreto reported 3,935 cases of *P. falciparum* and 21,048 cases of *P. vivax*, representing over 79% of all of the malaria cases reported in Peru during this time period [6]. Of the reported population, approximately 34.6% live in rural communities [7], where transmission is most intense [1]. Furthermore, because the major industries in the region include agriculture, fishing, logging and petroleum [1], a large portion of the population work as labourers that move between populated cities and large villages and rural areas with high malaria transmission to extract natural resources.

Previous studies have shown the existence of clusters of malaria transmission within populated communities and have suggested that focal “hot spots” of high malaria transmission may exist within areas with low levels of overall malaria incidence [3,8]. Recent research has also begun to investigate the implications of human travel in the movement of malaria from zones of high transmission to low transmission, identifying “sources” and “sinks” of malaria sporozoites [9]. Based on observed patterns of travel and transmission, it is suspected that similar processes may be occurring in the peri-Iquitos region. To better understand the heterogeneity of transmission in this area, this study sought to characterize vector capacity and malaria transmission risk in several suspected hot zones frequented by labourers and river travellers. It was hypothesized that these remote sites would demonstrate higher entomological inoculation rates (EIRs) than have been reported for the region. This study yields new insights into regional population movements that more accurately account for the higher than expected malaria rates in populated areas in the peri-Iquitos region.

## Methods

### Study sites and mosquito collections

**Figure 1:**
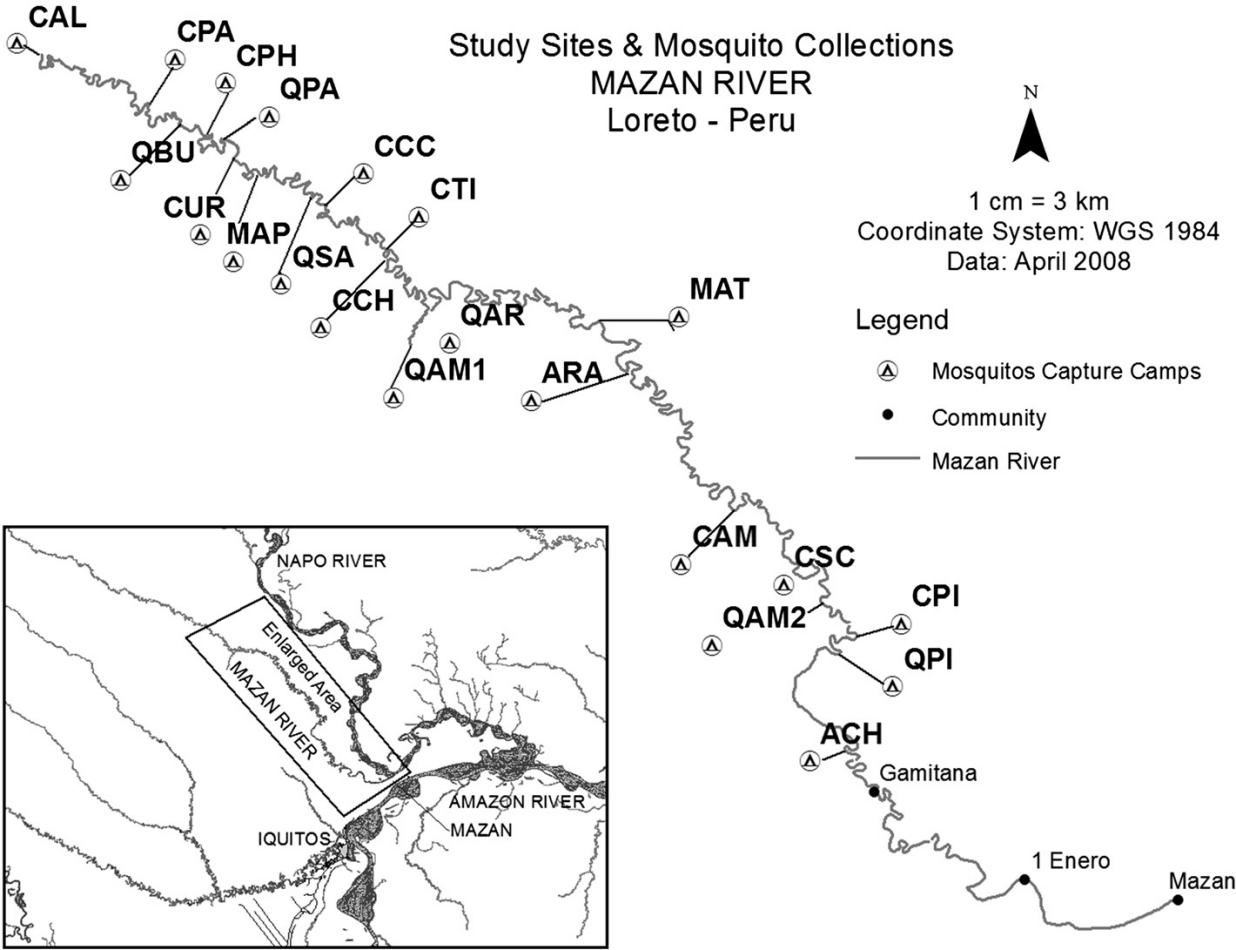
**Map of mosquito collection sites.** Mosquito collection sites are located approximately 40 to 50 km outside of the city of Iquitos. Nearby populated communities are included as points of reference for location, and site names and GPS coordinates are listed in Table 1.

Mosquito collections were conducted at 21 sites along the Mazan River in the Department of Loreto, Peru (Table 1 and Figure 1). The principal port of access is the village of Mazan, a community with a population of 5,800 located at the confluence of the Napo and Mazan Rivers. The sites are located approximately 40 to 50 km north-east of the city centre of Iquitos, the capital of Loreto, and are routinely accessible only by boat. From Mazan, inhabitants depart upriver throughout the year to fish, extract wood, hunt, and harvest palm leaves used in thatched roof construction in larger towns. Mosquito collections were conducted over the course of three survey periods, planned to simulate trips upriver that would be taken by local fishermen or loggers. Mosquito captures took place at riverside camps separated by expected travel distances using a 9–13 hp motor on a dugout canoe, known locally as a peke-peke, the typical mode of transportation in the area. The camps surveyed have no permanent residents but are known sites that are frequented by labourers and were selected for this reason.

**Table 1:** GPS coordinates for mosquito collection sites located along the Mazan River in Loreto, Peru

**Site code**	**Site name**	**Latitude**	**Longitude**	**Altitude (meters)**
**CAL**	Quebrada Calentura	−73°53’35.80”	−2°54’7.68”	104.428
**CPA**	Cocha Paña	−73°46’58.18”	−2°57’9.35”	106.351
**QPA**	Quebrada Paña	−73°46’17.08”	−2°57’30.05”	97.4586
**QBU**	Quebrada Bufeo	−73°46’17.12”	−2°57’57.11”	102.986
**CCC**	Cocha Camu Camu	−73°41’22.27”	−3°0’41.18”	111.157
**CUR**	Copal Urco	−73°45’1.71”	−2°58’46.85”	103.226
**MAP**	Campamento Mapillo	−73°43’58.46”	−2°59’17.47”	108.994
**QSA**	Quebrada Sara	−73°41’48.44”	−3°0’59.39”	110.917
**CCH**	Cocha Chiriyacu	−73°39’43.89”	−3°1’47.57”	104.188
**QAM1**	Quebrada Armas - Campamento Ajisal	−73°37’54.05”	−3°6’24.75”	105.63
QAR*	Quebrada Armas	−73°37’11.28”	−3°5’3.56”	104.188
**QAM2**	Punisique	−73°36’12.92”	−3°3’50.50”	102.025
MAT*	Mapa Tipishca	−73°28’13.19”	−3°6’19.59”	106.591
**ARA**	Arahuana	−73°29’3.27”	−3°7’18.57”	98.1797
**CTI**	Cocha Tigre	−73°39’17.85”	−3°2’ 2.73”	104.668
CAM*	Campamento 2/N	−73°24’28.32”	−3°13’3.72”	102.265
**CSC**	Campamento Suricaño	−73°21’19.65”	−3°16’12.83”	96.7377
**CPI**	Cocha Piurí	−73°19’26.29”	−3°18’4.77”	99.6216
**PCH**	Cocha Paña	−73°46’55.81”	−2°57’8.78”	103.226
QPI*	Quebrada Piurí	−73°19’23.03”	−3°18’11.10”	96.978
ACH*	Quebrada Achual	−73°19’35.96”	−3°23’27.91”	110.677

Codes marked with an asterisk (*) denote sites that were serially-visited during all three survey periods.

**Figure 2:**
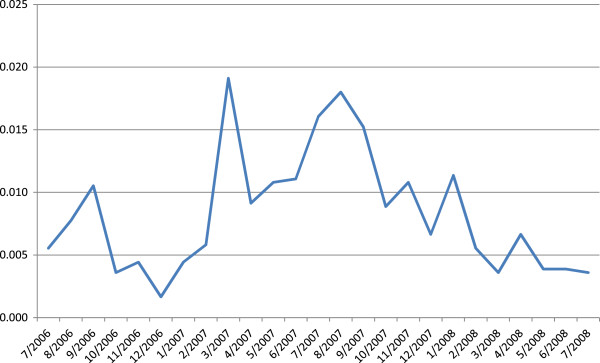
**Monthly *P. falciparum *Index (MFI) data for the community of Mazan.** MFI data from the community of Mazan for the year preceding the study was used to plan the timing of survey periods, allowing for the consideration only of recent infection rather than disease relapse. MFI is calculated as the number of reported *P. falciparum* cases per inhabitant per month.

The survey periods were separated by approximately four months each and situated at different points relative to seasonal peaks and troughs in the incidence of clinical cases of malaria in the region. The timing of each trip was based on monthly *P. falciparum* indices from the prior year (Figure 2) that marked April-May as a peak, December-February as a trough, and July-August as a midpoint. The first survey trip lasted 14 nights in April 2008, the second five nights in July-August 2008, and the third six nights in January-February 2009. Five collection sites were common across all three surveys, and 16 additional sites were visited only once.

Mosquitoes were collected from 1800–0700 by human landing capture and grouped by hour of collection and study site. Due to the low activity indices observed during the first survey period for the hours of 2200–0300 and 0600–0700, catches were abbreviated to 1800–2200 and 0300–0600 during the two subsequent survey periods. Collectors each exposed their lower legs and orally-aspirated landing mosquitoes, rotating shifts every two hours. The exact hours of collection and number of collectors varied slightly between nights, but were recorded to allow accurate calculation of person-hours spent collecting.

Collections were intradomiciliary and peridomiciliary if camps were inhabited. Where man-made shelters existed, they consisted of rudimentary structures with simple floors and roofs, lacking walls or an interior space separated from the outdoor environment. In cases where camps were not populated on the night that they were visited, collections took place at the most recent sites of human activity as demonstrated by the presence of campfire remnants, material that had been used in the of construction of camps, or other similar evidence of human habitation.

Specimens were separated and analysed by site and hour of capture. Upon return from each trip, female anophelines were identified to species in the laboratory using the morphological keys of Faran and Linthicum [10] and Consoli and Lourenço-de-Oliveira [11].

### ELISA analysis

Head and thorax sections of captured anophelines were individually separated from abdomens to avoid detection of pre-infective circumsporozoite protein present in midguts. Using the standard protocol distributed by the MR4 [12] and the specified monoclonal antibodies obtained from the Centers for Disease Control, mosquitoes were analysed individually in 96-well plates by enzyme-linked immunosorbant assay (ELISA) for the presence of *P. vivax* VK210, *P. vivax* VK247, and *P. falciparum* sporozoites. Results were read by an ELISA reader at 415 nm after 30 min and after 60 min, and the threshold value for positive results was set at twice the value of the negative control on each plate, according to Centers for Disease Control protocol.

### Malaria metric calculations

Based on the results from ELISA testing, sporozoite rates were determined for *P. falciparum*, *P. vivax* VK210, and *P. vivax* VK247 for each survey period, collection site and anopheline species. Hourly human biting rates for each site were calculated by dividing the number of mosquitoes caught by the number of person-hours spent collecting.

Because *Anopheles darlingi* composed over 98.5% of the mosquitoes collected and 97.4% of those that tested positive for sporozoites, all mosquitoes were treated as *An. darlingi* for the purpose of these calculations. Vectorial capacity index (VCI) and basic reproductive rate (BRR) were determined using the method outlined by Gilles [13]. VCI was calculated by multiplying the human biting rate by appropriate assumptions for bites per mosquito per night, the proportion of biting on humans (human blood index), and the expected longevity and expected infective life of the mosquitoes [13].

Biting rate per mosquito per night was set to 1/3 based on a gonotrophic cycle lasting three days for *An. darlingi* as observed in the Mato Grosso region of Brazil [13,14]. The human blood index for *An. darlingi* was assumed to be 0.59 based on previously published data from a rural region of the Brazilian Amazon [15]. Probability of survival of *An. darlingi* for one day was calculated from the parous rate [16]. The average parous rate for *An. darlingi* was reported to be 0.619 in the Upper-Maroni forest region of French Guiana [17], giving a probability of survival for one day of 0.852. Based on an average annual temperature of 28°C for the Loreto region [1], the sporogonic periods were conservatively estimated as eight days for *P. vivax* and 11 days for *P. falciparum* based on data from various experimental studies compiled and plotted against ambient temperature by Macdonald [18]. The probability of survival through the sporogonic period was calculated to be 0.172 for *P. falciparum* and 0.278 for *P. vivax*. The expected life of female *An. darlingi* was calculated as the negative reciprocal of the natural logarithm of the probability of survival for one day [13], giving a value of approximately 6.25 days.

BRR was calculated by multiplying VCI by appropriate assumptions for the susceptibility of the vector species to infection as determined by membrane feeds and the infective duration of an untreated human case calculated as the reciprocal of the human recovery rate [13]. Susceptibility of *An. darlingi* was conservatively assumed to be 0.410 for *P. falciparum* based on published data for membrane-fed *An. darlingi* caught in Belize [19] and 0.250 for *P. vivax* based on published data for membrane-fed *An. darlingi* caught in the Brazilian Amazon [20]. These susceptibility estimates fall just below the ranges observed in human-fed *An. darlingi* for *P. vivax* and in the lower half of the ranges for *P. falciparum*[21-24]*.* The recovery rate for *P. vivax* was assumed to be 1/9 based on factors used to mathematically model malaria transmission [25] and confirmed by data measured in a rural locality of Papua New Guinea [26]. The recovery rate for *P. falciparum* was assumed to be 1/200 based on case data compiled by Macdonald from several previous studies [27].

Nightly EIRs were calculated for each site by multiplying the measured sporozoite rates by the measured nightly human biting rates, and EIR values were compared across survey periods and collection sites.

### Malaria surveillance in the community of Mazan

The health post in the community of Mazan provides free, unrestricted diagnostics and treatment for malaria. Records were examined daily for the periods before and during the time that this study took place. To delineate transmission patterns and predict peak and trough months, monthly *P. falciparum* indices were used to consider clinical disease related only to recent infection rather than relapse.

### Active malaria surveillance among river travellers

Active surveillance for febrile illness and *Plasmodium* parasitaemia was conducted on the Mazan River during each of the three survey periods. Travellers encountered on the river in this region who gave informed consent were sampled by thick blood smear. Febrile patients were defined as symptomatic, while those lacking a fever at the time of sampling were considered to be asymptomatic. Rapid diagnostic tests (BinaxNOW Malaria Test; Binax, Inc, Scarborough, ME, USA) were performed on symptomatic individuals, and confirmed cases were treated according to standard protocols outlined by the Peruvian Ministry of Health. A total of 284 blood smears were collected, of which 188 were collected in April, 32 in July-August, and 64 in January-February.

### Statistical analysis

Measured sporozoite rates and human biting rates for the five serially-visited study sites were analysed using paired, two-tailed t-tests. Statistically significant differences between trips were defined as having *p*<0.05.

## Results

### Mosquito analysis

From July 2008 to April 2009, a total of 8,364 adult female mosquitoes were captured over 1,324 total person-hours, of which 8,347 (99.8%) anopheline mosquitoes were available for morphological species identification and analysis by ELISA for the presence of *P. vivax* VK210, *P. vivax* VK247, and *P. falciparum* sporozoites. Mosquitoes were collected from 21 locations along the Mazan River (Table 2). *Anopheles darlingi* comprised the majority collected (8,246, 98.58%), while six other species were collected in smaller quantities: *Anopheles oswaldoi* (40, 0.48%), *Anopheles mediopunctatus* (25, 0.30%), *Anopheles peryassui* (3, 0.04%)*, Anopheles nuneztovari* (2, 0.02%), *Anopheles kompi* (1, 0.01%) and *Anopheles shanonni* (1, 0.01%). Identifications to species of 47 (0.56%) mosquito specimens were unable to be performed due to morphological damage.

**Table 2:** Distribution of captured anopheline species at sites along the Mazan River in Peru

	***An. darlingi***	***An. mediopunctatus***	***An. kompi***	***An. nuneztovari***	***An. oswaldoi***	***An. peryassui***	***An. shannoni***	**Unidentified**	**Total**
**ACH**	539	0	0	0	5	0	0	13	557
**ARA**	386	0	0	0	2	0	0	0	388
**CAL**	188	1	0	1	1	0	0	1	192
**CAM**	1488	1	0	0	3	0	0	10	1502
**CCC**	88	1	0	0	0	0	0	0	89
**CCH**	48	0	0	0	2	0	0	0	50
**CPA**	160	0	0	0	1	0	0	0	161
**CPI**	272	2	0	0	0	0	0	0	274
**CSC**	644	0	0	0	1	0	0	3	648
**CTI**	8	1	0	0	0	0	0	0	9
**CUR**	68	0	0	0	0	0	0	2	70
**MAP**	253	0	0	0	0	0	0	1	254
**MAT**	2588	0	0	0	1	0	0	6	2595
**PCH**	85	0	0	0	0	0	0	0	85
**QAM1**	21	4	0	0	9	0	1	1	36
**QAM2**	136	4	0	0	0	0	0	5	145
**QAR**	310	0	0	0	9	0	0	2	321
**QBU**	103	0	0	0	0	0	0	0	103
**QPA**	9	0	0	0	1	0	0	0	10
**QPI**	834	9	1	1	4	3	0	1	853
**QSA**	18	2	0	0	1	0	0	1	22
**Total**	8246	25	1	2	40	3	1	46	8364
	(98.58)	(0.30)	(0.01)	(0.02)	(0.48)	(0.04)	(0.01)	(0.56)	

In the final row, the percent of total for each species is listed in parenthesis.

Of the specimens, 117 (1.4%) tested positive for sporozoites by ELISA. Of those that tested positive, 19 (0.23%) were positive for single infections of *P. falciparum*, 54 (0.65%) for *P. vivax* VK210, and 40 (0.49%) for *P. vivax* VK247. Four mixed infections were also detected: two (0.02%) for *P. falciparum* and *P. vivax* VK210, and two (0.02%) for *P. vivax* VK210 and *P. vivax* VK247. All mosquitoes positive for sporozoites were *An. darlingi*, with the exception of one positive *An. mediopunctatus* and one positive unidentified *Anopheles* spp (Table 3). The sporozoite rates for each *Plasmodium* species varied by capture location and survey period (Table 4). Human biting rates also varied between locations and survey periods, from 0.102 to 41.13 bites per person per hour (Table 5). Mean hourly human biting rates were 4.27, 17.61, and 3.40 bites per person per hour for April, July-August, and January-February, respectively. Hourly data from the April survey period showed a unimodal human biting rate that peaked at 2100–2200.

**Table 3:** Malaria positivity and sporozoite rate by ELISA antigen, anopheline species, and survey period

	**Number of positives (sporozoite rate, %)**			
**Survey period / Species**	***P. vivax* VK210**	***P. vivax* VK247**	***P. falciparum***	**Total number tested**
**April 2008:**				
*An. darlingi*	21 (0.521)	9 (0.223)	6 (0.149)	4034
*An. mediopunctatus*	0 (0.000)	0 (0.000)	0 (0.000)	2
*An. kompi*	0 (0.000)	0 (0.000)	0 (0.000)	0
*An. nuneztovari*	0 (0.000)	0 (0.000)	0 (0.000)	0
*An. oswaldoi*	0 (0.000)	0 (0.000)	0 (0.000)	4
*An. peryassui*	0 (0.000)	0 (0.000)	0 (0.000)	0
*An. shannoni*	0 (0.000)	0 (0.000)	0 (0.000)	0
Unidentified	0 (0.000)	0 (0.000)	0 (0.000)	9
**Total**	**21 (0.519)**	**9 (0.222)**	**6 (0.148)**	**4049**
**July-August 2008:**				
*An. darlingi*	0 (0.000)	1 (0.103)	1 (0.103)	967
*An. mediopunctatus*	0 (0.000)	0 (0.000)	0 (0.000)	7
*An. kompi*	0 (0.000)	0 (0.000)	0 (0.000)	0
*An. nuneztovari*	0 (0.000)	0 (0.000)	0 (0.000)	1
*An. oswaldoi*	0 (0.000)	0 (0.000)	0 (0.000)	16
*An. peryassui*	0 (0.000)	0 (0.000)	0 (0.000)	3
*An. shannoni*	0 (0.000)	0 (0.000)	0 (0.000)	0
Unidentified	0 (0.000)	0 (0.000)	0 (0.000)	5
**Total**	**0 (0.000)**	**1 (0.100)**	**1 (0.100)**	**999**
**January-February 2009:**				
*An. darlingi*	36 (1.116)	32 (0.992)	12 (0.372)	3227
*An. mediopunctatus*	0 (0.000)	0 (0.000)	1 (6.250)	16
*An. kompi*	0 (0.000)	0 (0.000)	0 (0.000)	1
*An. nuneztovari*	0 (0.000)	0 (0.000)	0 (0.000)	1
*An. oswaldoi*	0 (0.000)	0 (0.000)	0 (0.000)	20
*An. peryassui*	0 (0.000)	0 (0.000)	0 (0.000)	0
*An. shannoni*	0 (0.000)	0 (0.000)	0 (0.000)	1
Unidentified	1 (3.125)	0 (0.000)	1 (3.125)	32
**Total**	**37 (1.122)**	**32 (0.970)**	**14 (0.424)**	**3298**

Mosquitoes collected along the Mazan River were analyzed by enzyme-linked immunosorbant assay (ELISA) for *P. vivax* VK210, *P. vivax* VK247, and *P. falciparum*. Sporozoite rates (percentage of CSP positive mosquitoes) were calculated using the resulting number of positives.

**Table 4:** Malaria positivity and sporozoite rate by ELISA antigen, survey period, and collection site

	**Number of positives (sporozoite rate, %)**								
	**April 2008**			**July-August 2008**			**January-February 2009**		
**Site**	***P. vivax *VK210**	***P. vivax* VK247**	***P. falciparum***	***P. vivax *VK210**	***P. vivax* VK247**	***P. falciparum***	***P. vivax *VK210**	***P. vivax* VK247**	***P. falciparum***
**ACH**	0	1	0	0	0	0	0	0	0
	(0.000)	(0.510)	(0.000)	(0.000)	(0.000)	(0.000)	(0.000)	(0.000)	(0.000)
**ARA**	0	1	2	**-**	**-**	**-**	**-**	**-**	**-**
	(0.000)	(0.258)	(0.517)						
**CAL**	1	1	0	**-**	**-**	**-**	**-**	**-**	**-**
	(0.521)	(0.521)	(0.000)						
**CAM**	3	0	2	2	4	1	0	0	0
	(1.299)	(0.000)	(0.866)	(0.158)	(0.316)	(0.079)	(0.000)	(0.000)	(0.000)
**CCC**	0	1	0	**-**	**-**	**-**	**-**	**-**	**-**
	(0.000)	(1.124)	(0.000)						
**CCH**	0	0	0	**-**	**-**	**-**	**-**	**-**	**-**
	(0.000)	(0.000)	(0.000)						
**CPA**	5	2	0	**-**	**-**	**-**	**-**	**-**	**-**
	(3.106)	(1.242)	(0.000)						
**CPI**	0	0	1	**-**	**-**	**-**	**-**	**-**	**-**
	(0.000)	(0.000)	(0.365)						
**CSC**	**-**	**-**	**-**	**-**	**-**	**-**	0	1	0
							(0.000)	(0.154)	(0.000)
**CTI**	0	0	0	**-**	**-**	**-**	**-**	**-**	**-**
	(0.000)	(0.000)	(0.000)						
**CUR**	5	1	1	**-**	**-**	**-**	**-**	**-**	**-**
	(7.143)	(1.429)	(1.429)						
**MAP**	4	0	2	**-**	**-**	**-**	**-**	**-**	**-**
	(1.674)	(0.000)	(0.837)						
**MAT**	7	14	0	16	5	2	0	0	1
	(1.556)	(3.111)	(0.000)	(0.846)	(0.264)	(0.106)	(0.000)	(0.000)	(0.397)
**PCH**	1	1	0	**-**	**-**	**-**	**-**	**-**	**-**
	(1.176)	(1.176)	(0.000)						
**QAM1**	0	2	1	**-**	**-**	**-**	**-**	**-**	**-**
	(0.000)	(5.556)	(2.778)						
**QAM2**	1	3	1	**-**	**-**	**-**	**-**	**-**	**-**
	(0.690)	(2.069)	(0.690)						
**QAR**	2	5	1	0	0	1	0	0	0
	(0.858)	(2.146)	(0.429)	(0.000)	(0.000)	(1.316)	(0.000)	(0.000)	(0.000)
**QBU**	6	0	2	**-**	**-**	**-**	**-**	**-**	**-**
	(5.825)	(0.000)	(1.942)						
**QPA**	1	0	0	**-**	**-**	**-**	**-**	**-**	**-**
	(10.000)	(0.000)	(0.000)						
**QPI**	1	0	1	3	0	2	0	0	0
	(0.316)	(0.000)	(0.316)	(0.580)	(0.000)	(0.387)	(0.000)	(0.000)	(0.000)
**QSA**	0	0	0	**-**	**-**	**-**	**-**	**-**	**-**
	(0.000)	(0.000)	(0.000)						

A dash signifies that mosquito collection was not conducted at that site during that survey period. Mixed infections are considered to be positive for both antigens.

**Table 5:** Hourly human-biting rates of Anopheline mosquitoes collected in 20 sites along the Mazan River

	**Hourly human biting rate**		
**Site**	**April 2008**	**July-August 2008**	**January-February 2009**
**ACH**	4.261	6.500	1.265
**ARA**	12.516	-	-
**CAL**	3.310	-	-
**CAM**	5.500	27.522	0.102
**CCC**	2.697	-	-
**CCH**	1.389	-	-
**CPA**	3.833	-	-
**CPI**	6.683	-	-
**CSC**	-	-	13.224
**CTI**	0.273	-	-
**CUR**	1.061	-	-
**MAP**	4.704	-	-
**MAT**	10.488	41.130	5.143
**PCH**	4.250	-	-
**QAM1**	0.857	-	-
**QAM2**	4.028	-	-
**QAR**	5.548	1.652	0.245
**QBU**	2.641	-	-
**QPA**	0.313	-	-
**QPI**	10.533	11.239	0.408
**QSA**	0.489	-	-
***Average ± St. Dev.***	*4.269 ± 3.536*	*17.609 ± 16.356*	*3.398 ± 5.175*

For the five serially-visited sites, statistically significant (*p*<0.05) differences were observed between the April and January-February surveys for overall *Plasmodium* sporozoite rates and human biting rates and between the July-August and January-February surveys for overall *Plasmodium* rates only. No statistically significant differences were observed for sporozoite rates or human biting rates between the April and July-August surveys.

### Vectorial capacity index (VCI) and basic reproductive rate (BRR)

The VCI and BRR were calculated for each of the collection sites (Table 6). VCI values ranged from 0.035 to 14.080 for *P. vivax* and from 0.022 to 8.715 for *P. falciparum*. Mean VCI values for *P. vivax* were 1.461, 6.028, and 1.163 for April, July-August, and January-February (respectively), and 0.905, 3.731, and 0.720 for *P. falciparum*. As a measure of malaria transmission potential, for *P. vivax* 12 out of 20 (60%) sites in April, four out of five (80%) sites in July-August, and two out of six (33.3%) sites in January-February had VCI values greater than one, indicating vectorial conditions sufficient for sustained transmission. For *P. falciparum*, six out of 20 (30%) sites had VCI values greater than one in April, four out of five (80%) sites in July-August, and two out of six (33.3%) sites in January-February.

**Table 6:** **Vectorial capacity index and basic reproductive rate for *P. vivax *and *P. falciparum***

	**Vectorial capacity index (Basic reproduction rate)**					
	**April 2008**		**July-August 2008**		**January-February 2009**	
**Site**	***P. vivax***	***P. falciparum***	***P. vivax***	***P. falciparum***	***P. vivax***	***P. falciparum***
**ACH**	1.459	0.903	2.225	1.377	0.433	0.268
	(3.282)	(74.035)	(5.006)	(112.942)	(0.975)	(21.985)
**ARA**	4.285	2.625	-	-	-	-
	(9.640)	(217.476)				
**CAL**	1.133	0.701	-	-	-	-
	(2.550)	(57.519)				
**CAM**	1.883	1.165	9.421	5.832	0.035	0.022
	(4.236)	(95.566)	(21.198)	(478.207)	(0.079)	(1.773)
**CCC**	0.923	0.571	-	-	-	-
	(2.077)	(46.862)				
**CCH**	0.475	0.294	-	-	-	-
	(1.070)	(24.133)				
**CPA**	1.312	0.812	-	-	-	-
	(2.953)	(66.607)				
**CPI**	2.288	1.416	-	-	-	-
	(5.147)	(116.120)				
**CSC**	-	-	-	-	4.527	2.802
					(10.186)	(229.784)
**CTI**	0.093	0.058	-	-	-	-
	(0.210)	(4.739)				
**CUR**	0.363	0.225	-	-	-	-
	(0.817)	(18.429)				
**MAP**	1.610	0.997	-	-	-	-
	(3.623)	(81.730)				
**MAT**	3.590	2.222	14.080	8.715	1.761	1.090
	(8.078)	(182.242)	(31.680)	(714.667)	(3.961)	(89.360)
**PCH**	1.455	0.901	-	-	-	-
	(3.273)	(73.846)				
**QAM1**	0.293	0.182	-	-	-	-
	(0.660)	(14.893)				
**QAM2**	1.379	0.853	-	-	-	-
	(3.102)	(69.985)				
**QAR**	1.899	1.176	0.566	0.350	0.084	0.052
	(4.273)	(96.393)	(1.273)	(28.708)	(0.189)	(4.255)
**QBU**	0.904	0.560	-	-	-	-
	(2.034)	(45.889)				
**QPA**	0.107	0.066	-	-	-	-
	(0.241)	(5.430)				
**QPI**	3.606	2.232	3.847	0.140	0.201	0.086
	(8.113)	(183.023)	(8.657)	(195.287)	(0.314)	(7.092)
**QSA**	0.167	0.104	-	-	-	-
	(0.377)	(8.495)				

*P. vivax* here includes both the VK210 and VK247 antigens. A dash signifies that mosquito collection was not conducted at that site during that survey period.

Calculation of mean BRR values for *P. vivax* across all collection sites indicated 3.3, 13.6, and 2.6 secondary anopheline infections for each individual infected mosquito for April, July-August, and January-February (respectively). For *P. falciparum*, the mean BRR values for the three survey periods were 74.2, 306.0, and 59.0.

### Entomological inoculation rate (EIR)

Nightly EIRs were calculated for *P. vivax* and *P. falciparum* for each collection site and survey period (Table 7). *Plasmodium vivax* generally had higher nightly inoculation rates than *P. falciparum*, and, comparing individual sites across survey periods, the highest inoculation rates were generally observed in April, followed by July-August and January-February, though EIR values for the July-August survey exceeded those for the April survey in several instances. Average EIR values across all collection sites were 0.86, 0.91, and 0.02 infective bites per person per night for *P. vivax* and 0.19, 0.19, and 0.02 infective bites per person per night for *P. falciparum* in April, July-August, and January-February (respectively). Together, these give cumulative average inoculation rates of 1.04, 1.07, and 0.05 infective bites per person per night.

**Table 7:** **Nightly entomological inoculation rates by *Plasmodium *species, survey period, and collection site**

	**Nightly entomological inoculation rate**								
	**April 2008**			**July-August 2008**			**January-February 2009**		
**Site**	**PV**	**PF**	**Overall**	**PV**	**PF**	**Overall**	**PV**	**PF**	**Overall**
**ACH**	0.174	0.000	0.174	0.000	0.000	0.000	0.000	0.000	0.000
**ARA**	0.388	0.776	1.164	-	-	-	-	-	-
**CAL**	0.345	0.000	0.345	-	-	-	-	-	-
**CAM**	0.429	0.286	0.571	0.914	0.152	1.066	0.000	0.000	0.000
**CCC**	0.364	0.000	0.364	-	-	-	-	-	-
**CCH**	0.000	0.000	0.000	-	-	-	-	-	-
**CPA**	2.000	0.000	2.000	-	-	-	-	-	-
**CPI**	0.000	0.293	0.293	-	-	-	-	-	-
**CSC**	-	-	-	-	-	-	0.143	0.000	0.143
**CTI**	0.000	0.000	0.000	-	-	-	-	-	-
**CUR**	1.091	0.182	1.273	-	-	-	-	-	-
**MAP**	0.787	0.394	1.181	-	-	-	-	-	-
**MAT**	5.314	0.000	5.314	3.196	0.304	3.500	0.000	0.143	0.143
**PCH**	0.700	0.000	0.700	-	-	-	-	-	-
**QAM1**	0.571	0.286	0.857	-	-	-	-	-	-
**QAM2**	1.333	0.333	1.667	-	-	-	-	-	-
**QAR**	1.333	0.190	1.524	0.000	0.174	0.174	0.000	0.000	0.000
**QBU**	1.846	0.615	2.462	-	-	-	-	-	-
**QPA**	0.125	0.000	0.125	-	-	-	-	-	-
**QPI**	0.400	0.400	0.800	0.457	0.304	0.609	0.000	0.000	0.000
**QSA**	0.000	0.000	0.000	-	-	-	-	-	-

EIRs were calculated for *P. vivax* (PV), *P. falciparum* (PF), and total *Plasmodium* (overall). *P. vivax* here includes both VK210 and VK247. A dash signifies that mosquito collection was not conducted at that site during that survey period.

Of the mosquito collection sites, 17 out of 20 (85%) in April, four out of five (80%) in July-August, and two out of six (33.3%) in January-February had non-zero cumulative nightly inoculation rates. In April, eight sites had cumulative EIR values greater than one infective bite per person per night: Mapa Tipischa (MAT), Quebrada Bufeo (QBU), Cocha Paña (CPA), Punisique (QAM2), Quebrada Armas (QAR), Copal Urco (CUR), Campamento Mapillo (MAP) and Arahuana (ARA). Unprotected temporary inhabitants in these areas during the collection period would have received 5.3, 2.5, 2.0, 1.7, 1.5, 1.3, 1.2, and 1.2 infective bites each night, respectively.

### Malaria surveillance among river travellers

Analysis of thick blood smears collected from Mazan River travellers showed 17 positives of 284 total samples (5.99%). Broken down by survey period, 14 of 188 (7.45%) patients were positive in April, two out of 32 (6.25%) in July-August, and one out of 64 (1.56%) in January-February. Overall, only two out of 17 (11.76%) of those that tested positive for malaria were symptomatic, while the remaining 15 (88.24%) were asymptomatic at the time of sampling.

## Discussion

In this study, a remarkably high level of malaria transmission is demonstrated in a region previously characterized as hypo-endemic where village-based entomological studies have suggested very low EIRs. In contrast to such previous findings, EIRs comparable to those seen in holo-endemic regions of Africa have been observed in remote riverine sites tens to hundreds of kilometres away from larger human settlements, revealing a new level of heterogeneity of malaria transmission within the Peruvian Amazon. Furthermore, the existence of high rates of *Plasmodium*-infected *An. darlingi* in remote areas of human occupation suggests that anthropophilic mosquitoes with high vectorial capacity might be selected for, leading to asymptomatic, patent parasitaemia to maintain the malaria life cycle.

As a direct indicator of malaria risk for individuals temporarily residing in or travelling through this zone, EIR values were calculated for each of the campsites surveyed. Three previous studies also published EIR values for the peri-Iquitos region [28-30]. In an investigation of the effects of deforestation on vector biting rates in communities near to the road connecting Iquitos and Nauta, Vittor and colleagues found EIRs markedly lower than those found in this study with values ranging from 0.1 to 38 infective bites per person per year [28]. Another similar estimate of 10 to 20 infective bites per person per year, published by Roshanravan, was based on extrapolation of unpublished data [29]. A third study conducted by Reinbold-Wasson and colleagues surveyed several stably-inhabited sites along or nearby the Iquitos-Nauta highway, finding EIRs ranging from 0 to 60 infective bites per person per month during a period of historically high transmission (1996–7) [30]. As the only major estimates that had been published for the region, these values have been cited as an indication of malaria risk in the Peruvian Amazon.

Compared to the results of this study, however, it seems that these previously published values do not provide an accurate assessment of malaria transmission risk, markedly underestimating the true malaria burden in the peri-Iquitos region. This is likely due to the highly heterogeneous microgeographical nature of malaria. Though the sites examined by Vittor and her colleagues were located in a peri-urban area outside of Iquitos, these sites were selected due to varying levels of deforestation and not as areas of intense malaria transmission [28]. Similarly, Reinbold-Wasson and his colleagues surveyed sites along or nearby the Iquitos-Nauta road, unlike the undeveloped, transiently-populated camps surveyed here [30]. Correspondingly, the human biting rates found by Vittor and Reinbold-Wasson were much lower than the majority of those found in this study [28,30]. Furthermore, the EIR values published by Vittor and Roshanravan were based on estimated sporozoite rates of 0.5% rather than measured values found by mosquito capture and ELISA analysis, as have been conducted here [28,29]. Considering each survey period and site individually, nearly two-thirds of the localities surveyed in this study had sporozoite rates greater than this estimated value, reaching as high as 10.0%. This study provides the first assessment of malaria transmission risk and EIR in peri-urban hot zones in the Iquitos region based on direct measurement of sporozoite rates and hourly human biting rates, and the results are strikingly high compared to previous reports derived mostly from areas where sampling is more convenient from the population centre of Iquitos.

Several other studies have also investigated vector distribution and malaria transmission potential in Peru and other regions of the Amazon and can provide context for the results of this study. Compared to previously reported values for *An. darlingi* in the Loreto, Madre de Dios, San Martin, and Ucayali regions of Peru, sporozoite rates in the localities surveyed here were found to be 10- to 100-fold greater [31]. Human biting rates reported in Santa Clara, a community located outside of Iquitos, and in several localities in the region of Madre de Dios were substantially lower than the values reported for the top one-fifth to one-half of the sites surveyed here with the exception of one site in Madre de Dios that had values similar to the highest human biting rates reported in this study [32,33]. Notably, *An. darlingi* represented a much smaller proportion of the mosquitoes collected in each of these studies, exceeding 50% in only a minority of the localities they surveyed [31-33].

In general, the sites examined in this study were also among the highest in the Amazon in terms of malaria risk expressed as EIR. In the Brazilian Amazon, EIR values for *An. darlingi* have been reported at levels similar to those observed in this study; however, several of the sites surveyed here had EIR values up to twice as high as the greatest Brazilian values [34]. Such sites also had human biting rates and sporozoite rates that aligned with the top of the ranges that have been reported in Brazil [15,34-36]. As was observed in Peru, the species diversity in Brazil was different than that seen in this study, with *An. darlingi* comprising less than 50% of the mosquitoes collected in the majority of the localities surveyed in Brazil [15,34,36], which may suggest significant environmental differences. Studies from the Sifontes region of the Venezuelan Amazon and the Upper-Maroni Amazon region of French Guiana also report EIR values that are generally much lower than those reported in this study, while comparisons of human biting rates and sporozoite rates with these results vary by survey period [17,37]. In the Sifontes region of Venezuela, active vector control efforts at the time of collection were noted and mosquito collections were conducted indoors rather than outdoors, which may help account for the lower EIR values [37].

The study reporting results most similar to those reported here was conducted in the Ocamo region in southern Venezuela [38]. Mean cumulative EIR values in this region were comparatively higher than the averages observed in this study in January-February, but generally lower than those observed in April and July-August [38]. The same pattern can be seen for sporozoite rates and human biting rates [38]. Though the sites in Ocamo had permanent residents, these similarities may be related to other similarities in study sites, including the lack of insecticide-based vector control for 1–2 years prior to the study in Ocamo, the riverine location, and the high proportion of *An. darlingi*, which suggests ecological similarities [38].

In a broader sense, this study underlines the marked spatial heterogeneity of malaria transmission risk in the Peruvian Amazon. The importance of spatial heterogeneity of infection has been previously investigated in Peru and Brazil on a small scale within homes and between neighbouring homes, within gold mining areas, and within communities [3,8,39]. In the areas examined in these previous studies, at-risk zones were identified where risk of transmission for *P. falciparum* or *P. vivax* was significantly higher than in other zones of the areas surveyed [3,8]. Here, the existence of similar clusters of high transmission has been identified, but the spatial scale has been enlarged and the differences in transmission are much more pronounced. Of the 21 sites surveyed, nine sites had nightly EIR values greater than one infective bite per person per night during at least one of the survey periods, and the peak EIR value reached as high as 5.3 infective bites per person per night.

The results of this study also showed temporal heterogeneity in EIR and sporozoite rates across survey periods. In four out of the five serially-visited sites, the highest cumulative EIR values were observed in April and progressively declined for July-August and January-February. Only two of the six sites surveyed in January-February showed the presence of infective bites, and of those two neither showed an EIR value greater than 0.15 infective bites per person per night. A similar general trend can be seen for sporozoite rates, suggesting that malaria transmission in these hot zones, while intense, remains seasonally unstable.

Despite this instability, however, a large majority of the sites surveyed showed transmission potential, including several of those where malaria sporozoites were not found during one or more of the survey periods. In previous studies, mosquito behaviour has been regarded as a key determinant of malaria clustering [3,8]. In the Peruvian Amazon, *An. darlingi* has been identified as the most important regional vector, and its establishment in Loreto has been linked to the re-emergence and rapid growth of malaria in the peri-Iquitos region [1,15]. In this study, a dominant presence of *An. darlingi* was observed in every collection site across all three survey periods, including those where *Plasmodium* sporozoites were not detected. VCI values were found to be greater than one for *P. falciparum* or *P. vivax* in a significant portion of individual sites, indicating a strong potential for vector-based transmission. Moreover, although only one of the serially-visited sites showed VCI values greater than one for all three survey periods, several of the sites showing the highest trip-specific VCI values were not serially-visited. Together this data suggests that at least several of the sites surveyed in this study may be able to support stable malaria transmission for a large portion of the year, and, similarly, that the risk of developing intense transmission upon introduction of infective human hosts may exist in several of the sites where sporozoites were not detected during this study.

It is also important to consider the mechanisms sustaining transmission in these zones, which lack permanent human inhabitants. The greatest reported flight distance for *An. darlingi* is 7.2 km [40], making mosquito vector travel between sites unlikely. In a recent study, Wesolowski and her colleagues traced the distribution of malaria parasites through human movement, identifying various zones as “emitters” or “receivers” of malaria based on an evaluation of human travel networks [9]. It seems probable that frequent human travel for occupational purposes plays a major role in the establishment and maintenance of transmission in the sites surveyed in this study. Previous research has implicated the presence of asymptomatic malaria in maintaining malaria transmission in the Peruvian Amazon [3]. In this study, an overall rate of asymptomatic malaria of 5.28% was found amongst Mazan River travellers encountered in the region of the study sites. Although it is unclear whether these cases were contracted before or during travel in this region, the prevalence of parasitaemia, and particularly asymptomatic parasitaemia, is two- to three-fold greater than that seen in the community of Mazan, indicating that transmission is accelerated upriver.

Consideration of human travel also reveals the risk posed by zones of hyperendemicity, such as those considered in this study, for populated communities in the peri-Iquitos region. Labourers staying at the sites surveyed rarely employ malaria prevention measures (*eg* insect repellent or treated bed nets), and the sites themselves are undeveloped and have not been subject to vector control measures. Between June 2006 and July 2008, 2,697 malaria cases were detected in the community of Mazan. Considering these factors and the frequency of occupational travel, it seems likely that at least some of these cases were contracted in hot spot sites and carried back to travellers’ home communities. Previous research has shown that family members and neighbours living in the immediate vicinity of a human case are at higher risk for malaria exposure [3]. In this manner, human-related malaria importation enables intense hot zone transmission to affect more than just those who physically visit these sites by increasing disease risk for potentially large numbers of non-travelling inhabitants as well, likely helping to sustain malaria transmission in Mazan and other communities near Iquitos.

Ultimately, additional research is required to more precisely assess the impact of human travel between areas of hyperendemic transmission and populated communities. These hot spots correspond with sites that are frequented by labourers and travellers, providing challenges for disease control efforts in the surrounding communities to which exposed travellers return. Because of this, effective malaria control activities in the Peruvian Amazon should not only consider communities where malaria is present, but must also increase the preferential focusing of resources on populations frequenting areas of concentrated transmission and human travel. Doing so should reduce transmission in current hyperendemic zones, diminish the creation of confluence of transmission areas, and improve malaria control at the regional level.

## Conclusions

This study presents measures of vectorial capacity and indices of malaria transmission risk based on direct measurement of anopheline biting rates and sporozoite rates for several undeveloped, riverine campsites in the Peruvian Amazon, frequented by occupation-related travellers. The results shown here are significantly higher than those that have been previously published for the peri-Iquitos region, surpassing nearly all other published measures in the South American Amazon and reaching as high as values found in holo-endemic areas of Africa, while also exhibiting significant heterogeneity between collection sites and over time. These results further implicate human travellers as a potentially major and highly-mobile disease reservoir in the region, identifying a local target population involved in sporozoite movement and suggesting important considerations for malaria control efforts in populated communities to which malaria-carrying travellers return.

## Competing interests

The authors declare that they have no competing interests.

## Authors’ contributions

MK, PPY, MPO, RCG, DF, LCV, and HRF conceived and designed the study. MPO, PPY, BSP, KE, DF, SRP, RCG, WKP, CBC, and JMV acquired, analysed and interpreted data. BSP and MK drafted the manuscript. All authors read and approved the final manuscript.
